# Detecting Prolonged Stress in Real Life Using Wearable Biosensors and Ecological Momentary Assessments: Naturalistic Experimental Study

**DOI:** 10.2196/39995

**Published:** 2023-10-19

**Authors:** Rayyan Tutunji, Nikos Kogias, Bob Kapteijns, Martin Krentz, Florian Krause, Eliana Vassena, Erno J Hermans

**Affiliations:** 1 Donders Institute for Brain, Cognition, and Behaviour Radboud University Medical Center Nijmegen Netherlands; 2 Behavioural Science Institute Radboud University Nijmegen Netherlands

**Keywords:** biosensor, devices, ecological momentary assessments, experience sampling, machine learning, mental disorder, mental health, monitoring, physiological, prevention, psychological, smartwatches, stress, wearables

## Abstract

**Background:**

Increasing efforts toward the prevention of stress-related mental disorders have created a need for unobtrusive real-life monitoring of stress-related symptoms. Wearable devices have emerged as a possible solution to aid in this process, but their use in real-life stress detection has not been systematically investigated.

**Objective:**

We aimed to determine the utility of ecological momentary assessments (EMA) and physiological arousal measured through wearable devices in detecting ecologically relevant stress states.

**Methods:**

Using EMA combined with wearable biosensors for ecological physiological assessments (EPA), we investigated the impact of an ecological stressor (ie, a high-stakes examination week) on physiological arousal and affect compared to a control week without examinations in first-year medical and biomedical science students (51/83, 61.4% female). We first used generalized linear mixed-effects models with maximal fitting approaches to investigate the impact of examination periods on subjective stress exposure, mood, and physiological arousal. We then used machine learning models to investigate whether we could use EMA, wearable biosensors, or the combination of both to classify momentary data (ie, beeps) as belonging to examination or control weeks. We tested both individualized models using a leave-one-beep-out approach and group-based models using a leave-one-subject-out approach.

**Results:**

During stressful high-stakes examination (versus control) weeks, participants reported increased negative affect and decreased positive affect. Intriguingly, physiological arousal decreased on average during the examination week. Time-resolved analyses revealed peaks in physiological arousal associated with both momentary self-reported stress exposure and self-reported positive affect. Mediation models revealed that the decreased physiological arousal in the examination week was mediated by lower positive affect during the same period. We then used machine learning to show that while individualized EMA outperformed EPA in its ability to classify beeps as originating from examinations or from control weeks (1603/4793, 33.45% and 1648/4565, 36.11% error rates, respectively), a combination of EMA and EPA yields optimal classification (1363/4565, 29.87% error rate). Finally, when comparing individualized models to group-based models, we found that the individualized models significantly outperformed the group-based models across all 3 inputs (EMA, EPA, and the combination).

**Conclusions:**

This study underscores the potential of wearable biosensors for stress-related mental health monitoring. However, it emphasizes the necessity of psychological context in interpreting physiological arousal captured by these devices, as arousal can be related to both positive and negative contexts. Moreover, our findings support a personalized approach in which momentary stress is optimally detected when referenced against an individual’s own data.

## Introduction

Stress-related mental disorders such as major depression and anxiety disorders have gained increased recognition in the public eye. While a vast body of research exists regarding these disorders, studies have mostly focused on retrospective assessments of afflicted individuals. More recently, an increased interest has emerged in determining what makes some individuals more resilient to developing these disorders than others [1-4]. Investigating resilience, however, first requires an investigation of individual variation in stress reactivity before the development of psychological illness [1]. Following contemporary transactional frameworks of stress reactivity [5-7], such an approach would require the ability to assess how environmental or psychological stressors trigger biological and psychological responses depending on subjective appraisals of the degree to which an individual’s well-being is threatened. A strong motivation for this effort is the need to establish early warning signals for the onset of stress-related disorders. The ability to unobtrusively detect states of stress in daily life would enable early ecological interventions in those at risk by either flagging risk states to health-care providers or by delivering in-the-moment personalized interventions during these periods [8], thereby preventing or improving negative outcomes in patients [9], and ultimately reducing the societal and economic burdens of psychiatric illness on society [10].

Previous studies have used ecological momentary assessments (EMA) [11] to investigate stress reactivity in real life. These studies use repeated questionnaires (beeps) in daily life to investigate stress-related psychological processes [12-14]. Such methods used in stress-related disorders have identified real-life behavioral patterns that may explain or predict the onset of psychiatric illness [15,16]. They have also given insight into the effects of stress exposure on mood and its links to depression [17]. Despite providing substantial insights, these methods are often intrusive (ie, require active participation of patients), can lack feasibility in psychiatric populations, and may be influenced by careless responses or lower subjective insight into symptoms and associated states [18]. Furthermore, the sparse sampling of subjective states may miss time windows in which stressors occur. More recently, the reliability of such measures has also come into question, showing the importance of accounting for measurement errors [19]. Additionally, within the transactional model, the reliance on subjective assessments of stressors may also be conflated with the outcomes of interest that are being measured, such as mood measures that are often seen as indicators of mental health [20]. These issues indicate a growing need for novel and more reliable methods for passive and ambulatory mental-health monitoring.

The emergence of widely accessible wearable biosensors has raised the question of whether these devices can be used for ecological physiological assessments (EPA), either as an add-on or an alternative to EMA, in mental health monitoring. Wearable biosensors offer continuous recording of autonomic physiological markers such as skin conductance (SC) and heart rate (HR). These measures have been extensively validated in laboratory-based studies using controlled stress-induction protocols [21], showing increased HR and SC and decreased HR variability in response to stressors [22,23]. However, these autonomic physiological parameters are also associated with general arousal [24], including high-arousal states for positive affect [23]. Thus, using EPA may be more complicated in daily life than in the lab. While acute stress may trigger arousal, arousal itself may not necessarily signal the presence of stress. Both positive and negative affective states may thus be related to arousal measures [25]. The relationship of autonomic physiological responses to stressors in real life is not well understood. Some studies have attempted to investigate the physiology of daily life stress using scenarios or methods that are restrictive or burdensome [26,27]. For instance, a study using wearable biosensors could replicate lab findings to some extent [28]. However, this study lacked an environmental stressor and relied on the assumption that subjective stress measures can be taken as the “ground truth.” Overall reports of stressed states in this study were also relatively low when compared to the nonstress states. Finally, it did not allow probing the consequences of the accumulation of stress over a prolonged period, a key aspect when considering mental health. A recent review of the associations between subjective stress and HR measures also reflects these limitations, showing mixed results and inconsistency in findings [29].

**Figure 1:**
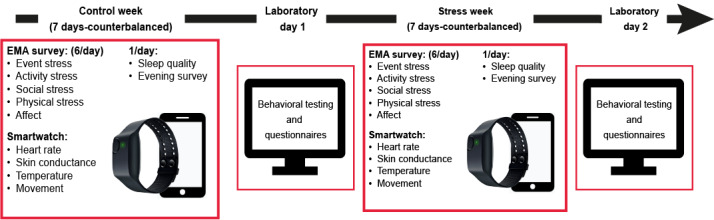
Study time line. Diagram portraying the sequence of participation in the study with counterbalanced weeks. Ecological momentary assessment (EMA) surveys included stress and affect assessments 6 times a day, in addition to sleep and evening questionnaires once a day. The wristband continuously measured physiological arousal, temperature, and movement.

To this end, we aimed to investigate the ability of active EMA measures, passive EPA monitoring, and the combination of these 2 methods to detect stress in real life. We investigated a population of first-year medical and biomedical students known to experience increased psychological distress [30]. Participants collected EMA and EPA data once during a week culminating in a high-stakes examination (ie, stress week) and another without (ie, control week). In line with the transactional framework of the stress response, this naturalistic experimental design allowed us to objectively manipulate prolonged stressor exposure while allowing EMA-based assessments of stress appraisals (through measures of event, activity, and social stress; see below) and separating these from EMA-based measures of mood reactivity as well as EPA-based measures of physiological reactivity to stressors. We first validated our protocol by testing between-week differences in EMA-based subjective appraisals of stress. We then assessed the impact of examinations and stress weeks on mood and physiology outcomes. Finally, we used individualized machine learning models to classify per time point (beep) which week participants were in using either mood, physiological, or a combination of both measures. This was done to investigate the utility of wearables as passive monitors of stress in ecologically relevant scenarios above mood-related EMA measures. We predicted increased autonomic physiological responses and negative affect and decreased positive affect in stress weeks. We expected that both EPA and EMA measures would successfully identify prolonged stress states and predicted that models combining EPA and EMA would outperform the single models. The study time line is shown in Figure 1.

## Methods

### Experimental Design

We recruited 84 right-handed, first-year bachelor’s students in the medical or biomedical science majors from Radboud Health Academy spanning 3 academic years (2017, 2018, and 2019). Participants were recruited through flyer distribution, presentations in common lecture halls, and web-based advertisements. A participant withdrew during testing, resulting in a total sample size of 83 participants used in the analysis. The programs were selected due to their structured examination weeks that occur every 5th and 10th week of a semester, allowing us to examine a period with higher stress levels during examination weeks as an ecological prolonged stressor. Right-handed participants were selected as participants were instructed to wear watches on their nondominant hand to reduce motion-related noise, with studies showing better signal from the left wrist than the right [31]. Only participants with no history of psychiatric illness were included in the study. Recruitment was stopped following the COVID-19 outbreak (March 2020).

Participants completed 2 weeks of EMA, one during an examination period (ie, stress week) and the other occurring on average 16 days (minimum=10; maximum=33) outside of these periods (ie, control week, demographics in Tables 1 and 2). We maintained at least one week between the end of one week and the start of the other to ensure sufficient recovery time from the stressor. Compliance rates were overall high, with 84% (70.56/84) of surveys completed within the allocated 1-hour window during both weeks. When accounting for missing and poor-quality physiology (EPA) data, completion rates dropped to between 76 and 77% (within ranges for other EMA studies) [32]. Compliance rates did not differ significantly between the weeks for either measure. Gender distribution was similar to that of students enrolled at the university (13739/24104, 57% female; according to the Radboud University website). We were unable to fully counterbalance the order of weeks due to the early termination of recruitment but instead controlled for it in all statistical analyses. Participants also filled out questionnaires and participated in magnetic resonance imaging sessions, which are outside the scope of this study and will be reported elsewhere.

**Table 1:** Descriptive statistics showing sex and program distributions, and week ordering.

Demographic items		Students, n (%)
**Sex, n (%)**		
	Female	51 (61.4)
	Male	32 (38.6)
**Course program, n (%)**		
	Medicine	61 (73.5)
	Biomedical sciences	22 (26.5)
**First week, n (%)**		
	Examination week	27 (32.5)
	Control week	56 (67.5)

**Table 2:** Completion rates for each of the examination and control weeks for both ecological momentary assessment (EMA) and ecological physiological assessment (EPA) measures.

Compliance Rates	Examination week			Control week		
	First quantile, n (%)^a^	Mean (%)	Third quantile, n (%)^a^	First quantile, n (%)^a^	Mean (%)	Third quantile, n (%)^a^
EMA	34 (81)	35.51 (85)	39 (93)	34 (81)	35.89 (85)	40 (95)
EMA with EPA	29 (69)	32.15 (76.55)	37 (88)	29 (69)	32.36 (77.05)	37 (88)

^a^First and third quantiles indicating 50% of participants had completion rates in the given range.

### Assessing Daily-Life Stress Through EMA and EPA

The comparison of the stress week (examination week) versus the control week allowed us to determine individualized patterns of stress reactivity. During these weeks, participants received 6 surveys a day at fixed intervals through SMS text message links. Participants were given a 1-hour window to fill out the surveys (like previous studies [33]). Individual surveys are referred to as beeps in the EMA literature. Surveys assessed different psychological aspects related to stress, including event, activity, social, and physical stress, as well as positive affect (PA) and negative affect (NA) outcomes. The first questionnaire of the day contained a sleep quality assessment, and the last included a self-reflection questionnaire. Participants were instructed to wear an Empatica E4 wristband (Empatica) recording ambulatory EPA data throughout both weeks (collected passively, continuously, and in the background). Participants were instructed to charge and synchronize the watch to researcher-specific accounts once a day for 1 hour. A detailed explanation was given to participants on the E4 operation with a practice session during the intake interview. The E4 devices collected blood pulse volume, electrodermal activity, 3-axis movement, and body temperature.

EMA surveys consisted of questions regarding subjective stress used for validating our experimental paradigm and mood questions (PA and NA) relating to our subjective outcome measures filled in on a 7-point Likert scale. Questions in the validation set probed four types of stress, as follows: (1) event-related stress assessed the most prominent event that occurred in between EMA beeps; (2) activity-related stress questions probed the activity participants were engaged in upon receiving the beep; (3) social-related stress addressed stress that may arise from the social context participants were present in (either being alone or with someone); and (4) physical-related stress was used as a control measure to account for environmental and physical demands. Mood outcome questions consisted of 4 items assessing positive mood and 5 items assessing negative mood based on the positive and negative affect schedule (PANAS), as validated in previous work [34]. EMA items on a reversed scale were first inverted. Items for each scale were summed to create a single score for each of the scales (ie, a single measure for event, activity, social, and physical stress). Total item scores were then rescaled, and a participant-centered measure was derived. Surveys that were not filled out within the assigned time window were excluded from further analyses. The same was done for outcome measure items relating to PA and NA.

EPA data cleaning was performed using Python (version 3.6.1; Python Software Foundation) [35]. Additional packages used for preprocessing included NumPy (version 1.18.1; Travis Oliphant) [36] and pandas (version 1.0.3; Wes McKinney) [37]. Time stamps for each survey instance were used to classify surveys as belonging to a stress or control week. Ten-minute time windows before each survey were selected for the extraction of physiology features acquired from the E4. Preprocessed interbeat interval (IBI) data were deemed too sparse to offer meaningful temporal domain analysis, with an average of 27% of IBIs successfully detected in our selected time window. This is within the margins of the manufacturer’s signal loss estimates for daily use. We instead selected average HR features from the resulting processed files from Empatica. The devices use a strict proprietary detection algorithm for the detection of IBIs, so these files can be used with minimal processing to derive global HR features. These features included the mean, minimum, and maximum HR. Raw SC was processed for offline use with the PyPhysio package (version 2.1; Andrea Bizzego) [38]. A minimum threshold of 0.01 µsiemens was set for the SC levels deemed of acceptable quality based on previous recommendations of a threshold between 0.01 and 0.05 µsiemens [39]. Data were first despiked to remove artifacts due to sudden hand motions using standard settings in the library. Data were then denoised to remove remaining artifacts through windowed filtering of changes in the signal greater than 0.02 µsiemens between subsequent samples. Additionally, an elliptic filter with a cut-off frequency set between 0.8 and 1.1 was applied to the data. SC data were subsequently deconvolved using a Bateman impulse response function into phasic and tonic components from which specific features were extracted (mean tonic activity, magnitude, area under the curve, and the number of phasic responses). The raw temperature measures were used to calculate the mean skin temperature as well as the slope as a function of change in skin temperature within the acquired time window. A total of 2 participants had a watch with faulty temperature sensors. These measures were substituted from the population mean and SD to avoid the loss of participants’ data due to missing data points in statistical models. The other sensors on this device were tested, and no errors were detected in other recordings. Finally, the root mean squared displacement in each time window was calculated from the accelerometer data. The extracted features were collected into a single data frame used for statistical analysis.

### Statistical Analysis

All statistical analyses were conducted in R (version 3.6.1, Ross Ihaka and Robert Gentleman) using generalized linear mixed effects models and random forest models (lmer and randomforest packages) [40,41]. Initial analyses examined overall differences in subjective stress between the 2 weeks to establish the validity of the experimental manipulation. We then tested for the effect of an examination week on affect and physiology. We additionally tried replicating previous findings associating momentary stress with physiology and mood. Mediation analysis was then used to explain the apparent differences in the relationships between the week type and momentary analyses. Additional covariates were added to all models. Covariates were selected to control for potential population differences and behavioral differences that may arise from being in an examination period. These covariates can be divided into subject-level, day-level, and beep-level covariates. Subject-level covariates modeled as fixed effects included sex, study program, and order of the weeks (ie, stress or control week first). Day-level covariates included the days relative to start (ie, day 1, day 2, day 3, etc), beep number, self-reported sleep duration, and the previous night’s alcohol consumption. Beep-level covariates modeled included hunger, caffeine intake, exercise, and sexual activity. Additionally, ambient temperature and accelerometer-derived movement were modeled for the EPA models. For further details regarding the surveys, code, and statistical modelling approaches see Multimedia Appendix 1 [42]

### Machine Learning Models

One of our goals was to assess the usability of ambulatory, nonintrusive measures to determine whether someone is currently in a stressed state. To this end, random forest models were used to determine the ability to classify whether participants’ beeps were in the stress or control week using the collected EMA mood and ambulatory EPA outcome data. Due to the subjective nature of mood items, participant-centered mood was used in all models. We conceptualized mood and physiology as outcomes of stressed states based on previous findings [17,43]. Individualized models were estimated using a Leave-One-Beep-Out (LOBO) approach at a single-participant level, where models were trained on individuals’ n-1 beep data and tested on the removed beep, repeating until all beeps had been removed. This is similar to the Leave-One-Trial-Out method used in other fields [44]. A total of 3 models were tested, as follows: Model 1 tested the ability to classify week type from (momentary) PA and NA, Model 2 from EPA data, and Model 3 from the combination of both. Models were tested against a bootstrap error distribution (n=10,000), with group effects tested using 2-tailed paired sample *t* tests against the mean subject-level bootstrap error. We tested the generalizability of the random forest models to a population level using a Leave-One-Subject-Out (LOSO) analysis in which models were trained on N-1 participants data set and tested on the removed participant, repeating until each participant had been removed once from the data set. Model predictions using the LOBO were then compared to those of the LOSO method to estimate the generalizability of machine learning models based on the data.

### Ethical Considerations

All procedures carried out were approved by the regional medical ethical review board (METC Oost-Nederland, protocol ID 2014-288). Written informed consent was obtained from all participants in Dutch following an intake interview where a detailed explanation of the procedures was carried out. Participants were given unique identifiers to maintain anonymity for all data acquired, with encrypted key files maintained by selected study personnel. In order to ensure the anonymity of the wearable data, participants were also provided with a study-specific account for data synchronization instead of personal accounts. Participants who completed all parts of the study were awarded 150 euros (US $158). The authors assert that all procedures contributing to this work comply with the ethical standards of the relevant national and institutional committees on human experimentation and with the Helsinki Declaration of 1975, as revised in 2008.

## Results

### Examination Periods are Associated With Increased Self-Reported Stress

**Figure 2:**
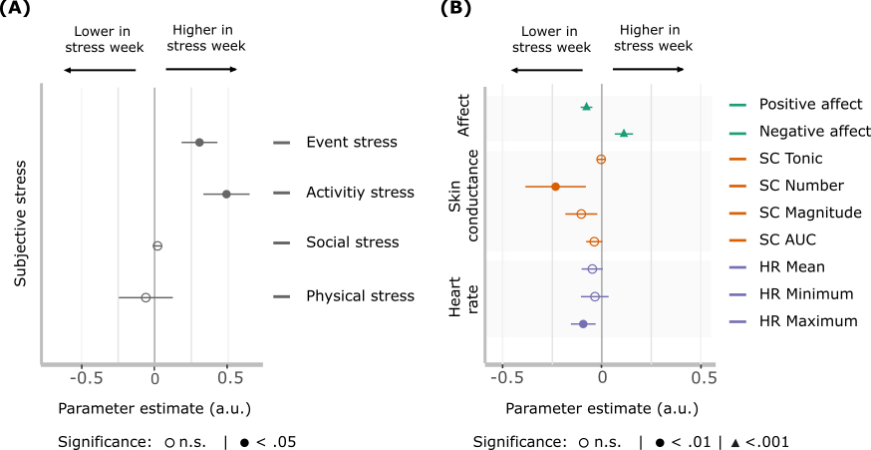
Fixed effects estimates of the between-week difference. (A) Event-related stress (pertaining to the most prominent event since the last survey) and activity-related stress (relating to the current activity participants are engaged in) are significantly higher in the stress week compared to the control week. (B) This is accompanied by increased negative affect, decreased positive affect, and decreases in averages of multiple arousal-related physiological measures. Error bars represent 95% CI. AUC: area under the curve; HR: heart rate; SC: skin conductance.

We found a significant increase in prominent stressful events (ie, event-related stress, β=.30; 95% CI 0.18-0.42; *P<*.001) and current reports of stress (ie, activity-related stress, β=.51; 95% CI 0.30-0.71; *P<*.001) in the examination stress versus control week. Social stress was not significantly different between the 2 weeks. The control items measuring physical stress also did not differ significantly between the weeks, showing that increases in subjective stress were likely due to our experimental manipulation instead of environmental or physical changes (Figure 2A, Table S1 in Multimedia Appendix 1). As anticipated, not all beeps in stress weeks were subjectively reported as stressful, while some beeps during the control week were subjectively rated as stressful. To quantify this, subjective stress variables for event, social, and activity stress were aggregated across both weeks. A median split was then used to estimate the percentage of incongruent self-report beeps (ie, false positives in stress weeks and false negatives in control weeks). On average, across participants, 45% (2157/4794) of the beeps yielded self-reported stress incongruent with the week type. Machine learning models using self-reported stress assessments in a LOBO approach to classify week types achieved similar error rates, with 43% (2016/4794) of beeps being classified as the wrong week type.

In accordance with our expectations, we also saw an increase in NA (β=.12; 95% CI 0.08-0.17; *P_fdr_*<.001), and a decrease in PA (β=–.08; 95% CI –0.11 to –0.05; *P_fdr_*<.001) during the stress week (Figure 2B). Unexpectedly, we found a decrease in physiology arousal-related measures during the examination week, including the number of SC responses (log-mean –0.27; 95% CI –0.42 to –0.12; *P_fdr_*<.001), and maximum HR (β=–.10, 95% CI –0.16 to –0.03; *P_fdr_*=.003). Figure 2B, Table S2 in Multimedia Appendix 1.

### Momentary Subjective Stress is Associated With Mood and Physiology

**Figure 3:**
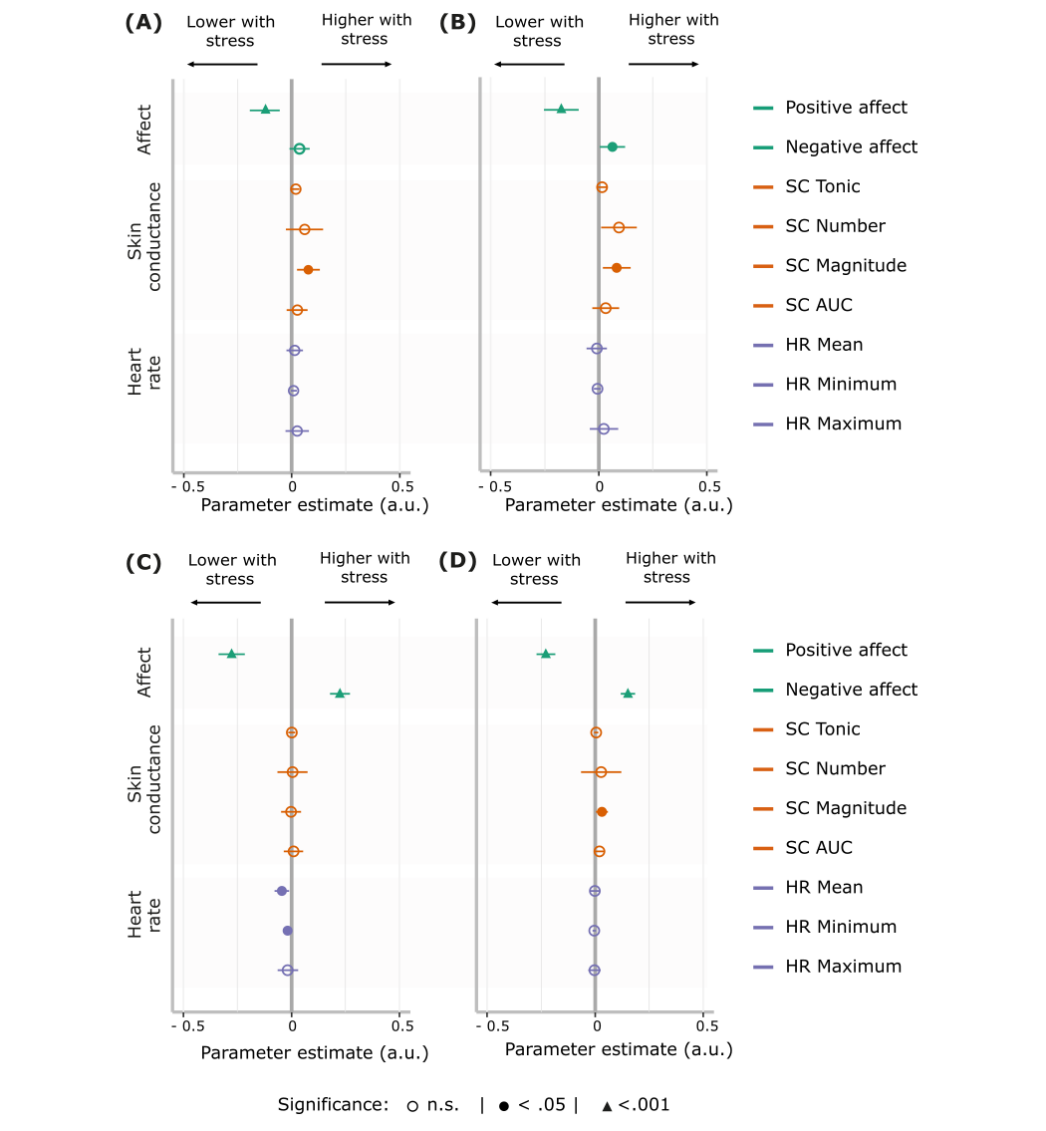
Effect estimates for the associations between within-beep fluctuations in subjective stress and measures of mood and physiology. (A) Event-related, (B) activity-related, (C) social-related, and (D) physical-related stress are generally associated with a decrease in positive affect, an increase in negative affect, and increases in some of the measures of physiological arousal. *P* values are corrected for multiple comparisons using false discovery rate corrections. Error bars represent 95% CIs. AUC: area under the curve; HR: heart rate; SC: skin conductance.

To explore the dynamics underlying the unexpected average decrease in measures of physiological arousal during the stress week, we investigated the link between in-the-moment fluctuations in subjective stress and outcome measures (ie, within the same beep mood and physiological arousal). We found a positive association between NA and activity-related (β=.06; 95% CI 0.01-0.12; *P_fdr_*=.05), social (β=.22; 95% CI 0.18-0.27; *P_fdr_<*.001), and physical stress (β=.15; 95% CI 0.12-0.18; *P_fdr_*<.001). The opposite was true for PA for event-related (β=–.12; 95% CI –0.19 to –0.06; *P_fdr_*<.001), activity-related (β=–.17; 95% CI –0.25 to –0.09; *P_fdr_<*.001), social (β=–.28; 95% CI –0.34 to –0.22; *P_fdr_*<.001), and physical stress (β=–.23; 95% CI –0.27 to –0.18; *P_fdr_* <.001). The magnitude of SC responses was associated with activity (β=.08; 95% CI 0.02-0.15; *P_fdr_*=.02), event (β=.07; 95% CI 0.02-0.13; *P_fdr_*=.02) and physical stress (β=.03; 95% CI 0.00-0.06; *P_fdr_*=.04). For HR measures, mean (β=–.04; 95% CI 0.08 to –0.01; *P_fdr_*=.04) and minimum (β=–.02; 95% CI –0.03 to –0.00; *P_fdr_*=.01) HR were negatively associated with social stress. Thus, within-beep fluctuations in subjective stress are associated with expected mood changes and increases in physiological arousal (Figure 3, Table S3 in Multimedia Appendix 1).

### Positive Mood is Related to Increased Arousal and Mediates Week Changes

**Figure 4:**
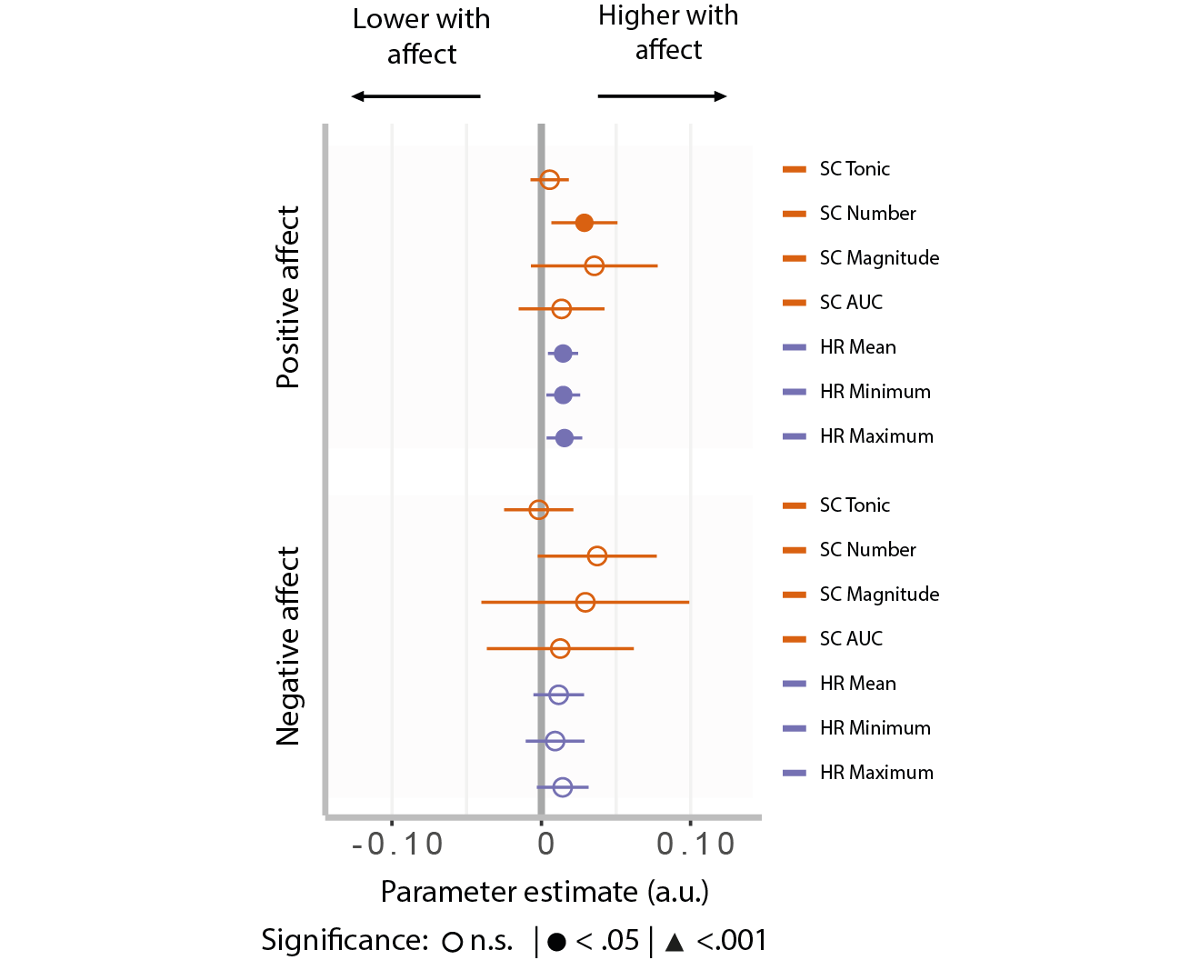
Relationship between momentary affect and physiology. Arousal-related physiological measures (magnitude of skin conductance responses and mean and minimum heart rate) were linked to positive affect but not to negative affect. *P* values are corrected for multiple comparisons using false discovery rate correction. Error bars represent 95% CI. AUC: area under the curve; HR: heart rate; SC: skin conductance.

To investigate whether the observed decreases in physiological arousal during stress weeks could instead be linked to reduced PA, we tested the within-beep association between affect and physiological arousal. Increased PA was related to increase in the number of SC responses (β=.08; 95% CI 0.02-0.06; *P_fd_*_r_=.04), and mean (β=.01; 95% CI 0.001-0.02; *P_fdr_* =.01), minimum (β=.01; 95% CI 0.001-0.02; *P_fdr_*=.03), and maximum HR (β=.01; 95% CI 0.001-0.02; *P*_fdr_=.03; Figure 4, Table S4 in Multimedia Appendix 1). Thus, in addition to subjective stress, PA is also positively associated with momentary physiological arousal.

Next, to confirm that the observed average decrease in physiological arousal observed during the stress weeks is due to the decrease in PA, we assessed whether PA statistically mediated the effects of week type on reductions in physiological arousal. We specifically focused on the arousal measures linked to subjective stress and PA: the number of SC responses and their magnitudes. Results indicated that PA significantly mediated the relationship between SC magnitude and week type (7.3%, mediating estimate=–0.013; 95% CI –0.03 to 0.00; *P*=.03) but not fully (direct estimate=–0.166; 95% CI –0.23 to –0.10; *P<*.001), indicating potential additional mechanisms are at play. The effect of week type on the number of SC responses was not mediated by PA.

### Machine Learning Classification of Beeps Using Mood and Physiology

**Figure 5:**
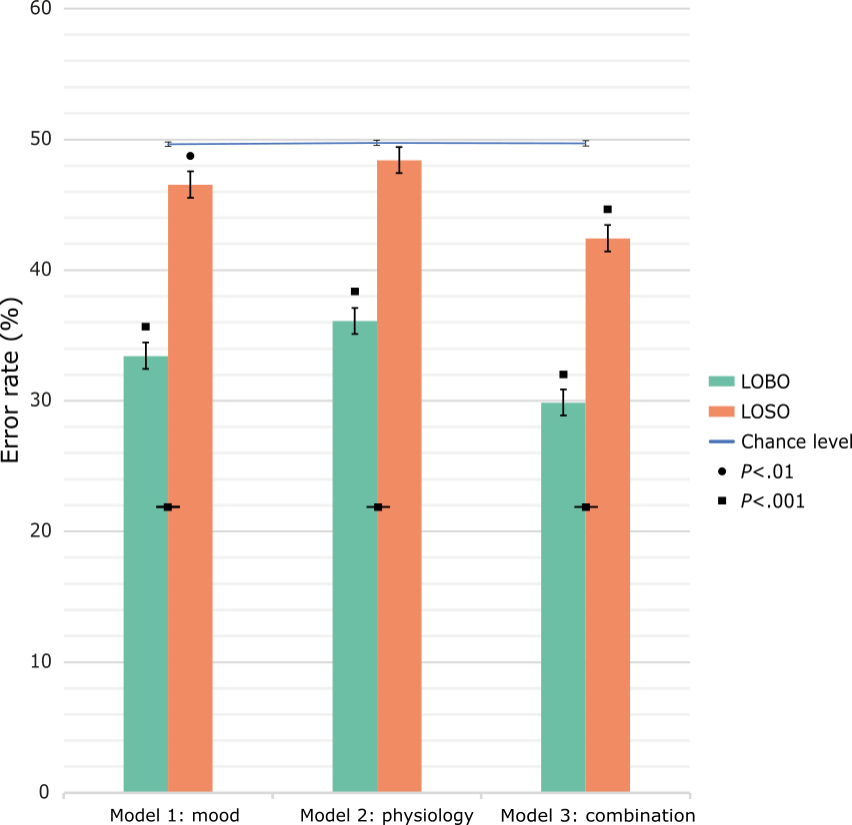
Random-forest classification error estimates. Average error estimates and error bars (representing the SE of the mean) for each of the random forest models. Combinations of mood and physiology yield superior classification, and individually trained and tested models (Leave-One-Beep-Out [LOBO]) perform better than models trained on group-level data (Leave-One-Subject-Out [LOSO]). Chance levels estimated from the permutation test and 95% CI are shown in blue. Significance levels between bars indicate between model comparisons, and above bars indicate model comparisons to chance levels. *P* values are corrected for multiple comparisons using false discovery rate.

We next examined to what extent prolonged stress (ie, stress vs control week) can be classified from individual beeps using machine learning based on affect, physiological arousal, or a combination of both using individualized LOBO models. The mean subject-level error was 33.45% (SD 2.21%) for Model 1 (based on affect), 36.11% (SD 2.72%) for Model 2 (based on physiology), and 29.87% (SD 3.45%) for Model 3 (based on affect and physiology combined). Hence, the combined model outperformed the single-variable models. All models performed significantly above chance on an individual level for all but one subject (Figure 5, Multimedia Appendix 1). Group-level effects were further tested with 2-tailed paired-samples *t* tests with FDR correction comparing the LOBO models to the mean bootstrapped error. Model 1 (affect, mean_diff_ –16.29; t_80_=–64.06; *P*_fdr_<.001), Model 2 (physiology, mean_diff_ –13.87; t_78_=–50.38; *P*_fdr_<.001), and Model 3 (combination, mean_diff_ –19.45; t_78_=–48.94; *P*_fdr_<.001) all performed above chance. Paired-samples, 2-tailed *t* tests comparing the within-participant error rates between the LOBO models showed that Model 3 (ie, combined EMA and EPA) outperformed Model 1 using mood alone (mean_diff_ 3.64; t_78_=19.20; *P<*.001), which in turn outperformed Model 2 using EPA alone (mean_diff_ 2.60; t_78_=14.65; *P<*.001). While overall the EMA mood models performed better, for some participants, models 1 and 2 had almost equivalent performance.

### Individualized Models Offer Better Predictions Than Group-Based Models

We next investigated the generalizability of these models from an individualized approach to a population-level one (using group-level classification) through LOSO cross-validation. M1 using affect (45.85%, SD 9.50%), M2 using physiology (48.42%, SD 8.05%), and M3 using the combination (42.44%, SD 9.00%) were again tested against their bootstrapped counterparts.

For some individual participants, LOSO models performed significantly above chance level (Model 1-affect n=45, 54.1%; Model 2-physiology n=30, 37.9%; and Model 3-combination n=55, 69.6%) in classifying week type (Multimedia Appendix 1). Group-level analysis using a 2-tailed paired sample *t* test showed that only model 1 (affect, mean_diff_ –3.53; t_80_=–3.59; *P*_fdr_=.005), and model 3 (combination, mean_diff_ –6.55; t_78_=–6.81; *P*_fdr_<.001) performed better than chance. Model 2 did not perform above chance (physiology, mean_diff_ –1.34; t_78_=–1.54; *P*_fdr_>.99).

We additionally directly compared the classification errors between the individualized and group models for each participant using a paired-sample *t* test with FDR correction. All individualized LOBO models performed better than the group-level LOSO models. LOSO Model 1 (mood) performed significantly worse than the equivalent LOBO model (mean_diff_ 11.43; t_80_=12.17; *P*_fdr_<.001). LOSO Model 2 (physiology) was also significantly worse than the LOBO counterpart (mean_diff_ 8.11; t_78_=8.61; *P*_fdr_<.001). LOSO Model 3 (combination) similarly performed worse than the LOBO model counterpart (mean_diff_ 12.61; t_78_=11.74; *P*_fdr_<.001). In sum, individual models vastly outperformed group-based models.

## Discussion

This study investigated physiological and psychological responses to ecological stressors in daily life (ie, examination weeks in students) to determine the usability of passive monitoring technologies for detecting prolonged stress. We used EMA and EPA to track subjective stress, mood, and arousal-related physiology. Our findings confirmed an overall increase in subjective stress during examination weeks. As hypothesized during the stress week, NA increased and PA decreased. Contrary to what was expected, lower SC and HR arousal measures were recorded during the stress week. At a beep-to-beep time scale, increased subjective stress was associated with increased NA, decreased PA, and increased SC responses. Interestingly, PA was also associated with increased SC responses and partially mediated the between-week differences in SC we found. Thus, the observed decreases in physiological arousal measures were (at least partially) due to a reduction in PA. Using a machine learning approach, we showed that the combination of individual mood and physiology was best able to detect whether individual beeps stemmed from stress or control weeks. We conclude that passive monitoring with wearable biosensors can detect prolonged stress, highlighting the importance of mood measures to dissociate positive and negative arousal.

In line with previous work, the stress week resulted in increases in self-reported stress, validating our paradigm [45]. We observed expected changes in mood, with increased NA and decreased PA. However, arousal measures were surprisingly reduced during the stress week. The observed overall decrease in physiological arousal during stress weeks appears at odds with the positive association between within-beep subjective stress and increased arousal in our analysis and previous works [22,26,28]. This finding reveals a dissociation between prolonged and acute stress. While prolonged stress leads to increased within-beep peaks in self-reported acute stress, it also results more generally in decreased PA and decreased overall average arousal. Our results suggest that reduced arousal may be linked to reduced PA seen in the stress weeks (irrespective of peaks in subjective stress). Our mediation analysis corroborates this mechanistic link, confirming that PA partially mediates the effect of week type on reduced arousal. While this may seem counterintuitive, SC and HR measures are known to respond to both positive and negative events, showing that physiological arousal is not valence specific [23,46]. This fits with a recent review observing the most consistent link between high-arousal subjective states and increased HR measures [29]. Within a theoretical framework of affect dynamics, these findings also align with the circumplex theory of emotion and valence, linking the 2 on a grid-like schema of valence and arousal [47]. Thus, the net effect of prolonged stress exposure stems from a reduction in overall arousal driven by reductions in positive mood that persist outside of peak moments of acute stress.

We subsequently tested the ability of machine learning models to classify individual beeps as stemming from stress or control weeks with physiology, mood, or a combination of both. Physiology models could classify beeps almost as well as mood models (3.85% difference on average). However, and more importantly, combination models showed the highest accuracy. Hence, the addition of mood questions to physiological arousal provides valuable information for prolonged stress detection. This converges with the mixed models and mediation results: accounting for valence through mood is necessary to distinguish stress-induced from PA-induced arousal. Our findings provide a mechanistic explanation for why previous studies using SC trigger-based EMA to detect stress captured positive arousal instead [46]. In addition to demonstrating that affect and arousal offer better than chance classification levels, we also show that they achieve higher accuracy in classifying week type than classification based on a median split across explicit subjective stress measures. Using a combination of EPA and mood EMA may also reduce issues related to measurement errors seen within EMA. Additionally, assessing mood and physiological arousal may offer a more nuanced measure of stress states that is not dependent on activities or events that occurred since the previous beep. This approach is also common in laboratory research on stress, where mood questionnaires and physiological arousal measures are often used to quantify stress [48]. In sum, combining a wearable biosensor with minimally invasive mood assessment might offer the best approach to detecting stress in both healthy and clinical populations, offering a more feasible approach than full EMA batteries.

Besides demonstrating the utility of physiological monitoring, our results highlight the importance of individualized approaches in stress detection. Classification models trained and tested on individuals’ own data (LOBO) performed significantly better than those trained on group data (LOSO). Our individualized approach offers drastic improvements in the classification of stress states in comparison with group approaches [28]. This supports findings in previous work, where large between-participant differences in dynamic ranges of responses limited applications of machine learning at the population level, pointing toward the need for individualization [28]. Intuitively, the same experience can generate different physiological and psychological responses in different individuals based on a multitude of factors, such as sex, appraisal, or clinical traits. For example, patients with anxiety may display a very different physiological response to stress than those with depression (hyper- vs hypoactivation) [49]. This is a key strength of the current approach, fully aligned with recent developments in personalized psychiatry: individualized models allow for greater prediction accuracy than a one-size-fits-all approach.

Worth noting is that classification accuracy of our machine learning models was relatively low in this study compared to many other ML studies. Previous studies found limited applicability of such algorithms due to variance in ranges of physiological responses between participants [28]. However, by using a within-participant design, we circumvent such issues. Importantly, lower accuracy stems from the ecological design: our models did not classify weeks but rather individual beeps within the weeks (approximately 70% of beeps were correctly assigned to the weeks in the best models). Through the median split of our data based on self-reported stress, we clearly demonstrate that even during a stress week, participants are not stressed 100% of the time. This was an intentional design choice: the goal was to test the ability of physiology and mood measures combined to detect momentary stress states during heightened periods of stress, which may be required, for example, for detecting warning signals. Furthermore, the accuracy achieved with our real-life models is also on par with more recent laboratory studies classifying affect from wearables and infrared cameras [50]. Hence, the classification accuracy found in this study represents what might occur in the general population during real-life stress periods (including stressful moments in regular weeks and regular moments in stressful weeks) and supersedes directly asking about stress.

This study provides early evidence for the successful detection of prolonged periods of stress in individuals. However, some limitations warrant discussion. This study was purely cross-sectional, meaning that we are unable to make connections in how our findings can be used for predicting mental health outcomes and resilience in the long term. Prospective and longitudinal designs with explicit measures of resilience are needed for this. This is also important within the context of promoting resilience, as prospective detection of vulnerability is the next step in this line of research, with the aim of identifying early warning signals [51]. More research (some of which is currently underway [52]) will be needed to extend these results into prospective stress detection algorithms [49]. Additionally, it is important to consider the reliability and validity of the devices used in this study. Previous research has shown that these devices offer reliable measures of SC for stress detection [53,54], but this may not extend to daily life scenarios, which are inherently noisier [55]. While we cannot eliminate noise from our data, preprocessing steps such as despiking and filtering allowed us to derive a cleaner signal. Additionally, the inclusion of an accelerometer-derived motion component in our models can also partially explain variance related to wrist displacement. Furthermore, we refrain from including HR variability metrics in our analysis for this specific reason, and such measures, while more specific to sympathetic nervous system activity, are also more susceptible to noise.

It may also be argued that the uncontrolled nature of the study is a detriment to the findings and has an impact on the reliability of the proposed measure. However, the ecological validity of this study is rather a strength in providing a necessary translation of laboratory measures to a real-life setting [56]. Additionally, we controlled for several potential confounds that may impact the reliability of our measures, such as those differences in behavior across weeks (ie, alcohol intake, sleep, caffeine, and exercise). Finally, and worth noting, this study focused on a relatively smaller sample of students, which may limit generalizability to other contexts. However, we also note that an examination stressor may resemble many real-life stressors and daily hassles, such as work deadlines. Yet, these results may not generalize to more severe, traumatic, and stressful life events. Acute stressful events may lead to very different arousal responses, and future research is needed to address this topic. However, having to rely on the occurrence of such events in a study may prove difficult and would require longer periods of assessment in the hopes of capturing these types of acutely stressful moments. Indeed, this is an issue that has already been addressed by previous attempts at classifying stress from such devices [28].

In conclusion, this study shows that EPA may be used for monitoring stress-related mental health but highlights the importance of affect ratings to dissociate changes in arousal due to stress versus PA. A combination of physiology and mood measures is optimal for detecting prolonged stress, and personalized approaches to modeling these variables are necessary. If successfully implemented at a wider scale, our findings may have implications for disease prevention, potentially helping to reduce the overall disease burden of stress-related disorders through personalized early-warning systems and treatment strategies.

## Appendix

Multimedia Appendix 1 Supplementary text file with extra details on code and analysis.

## Abbreviations

EMA: ecological momentary assessment
EPA: ecological physiological assessment
HR: heart rate
IBI: interbeat interval
LOBO: Leave-One-Beep-Out
LOSO: Leave-One-Subject-Out
NA: negative affect
PA: positive affect
PANAS: positive and negative affect schedule
SC: skin conductance

## Data Availability

The data sets generated during and/or analyzed during this study are not publicly available due to the sensitive and potentially identifiable nature of the data but are available from the corresponding author on reasonable request. Data repository also includes all scripts used in preprocessing and statistical analysis, which are also included on the GitHub repository linked in Multimedia Appendix 1.
